# Artemisinin upregulates neural cell adhesion molecule L1 to attenuate neurological deficits after intracerebral hemorrhage in mice

**DOI:** 10.1002/brb3.2558

**Published:** 2022-03-29

**Authors:** Jianjiang Wang, Jie Yin, Xi Zheng

**Affiliations:** ^1^ Department of Neurosurgery General Hospital of Xinjiang Military Region Urumqi China

**Keywords:** artemisinin (ART), intracerebral hemorrhage (ICH), neural cell adhesion molecule L1, oxidative stress, inflammation

## Abstract

**Background and purpose:**

Intracerebral hemorrhage (ICH) is a subtype of stroke and results in neurological deficits in patients without any effective treatments. Artemisinin (ART), a well‐known antimalarial Chinese medicine, exerts multiple essential roles in the central and peripheral nervous system due to its antioxidative and anti‐inflammation properties. Neural cell adhesion molecule L1 (L1CAM, L1) is considered to be implicated in neural development, functional maintenance, and neuroprotection during disease. However, whether these two essential molecules are neuroprotective in ICH remains unclear.

**Methods:**

Therefore, the present study investigated the influence of ART on the recovery of neurological deficits in a mouse model of ICH induced by collagenase and the underlying mechanism.

**Results:**

It was revealed that ART is capable of upregulating L1 expression to alleviate brain edema, reduce oxidative stress, and inhibit inflammation to alleviate ICH‐induced brain injury to improve the neurological outcome in mice suffering from ICH.

**Conclusion:**

These results may lay the foundation for ART to be a novel candidate treatment for ICH.

## Graphical abstract

We revealed that ART is capable of upregulating L1 expression to alleviate brain edema, reduce oxidative stress, and inhibit inflammation to alleviate ICH‐induced brain injury to improve neurological outcome and in mice suffering from ICH. These results may lay the foundation for ART to be a new candidate treatment for ICH.

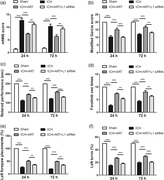

## 1 INTRODUCTION

Intracerebral hemorrhage (ICH), a stroke subtype induced by vascular rupture, is well known for its high rates of mortality and morbidity (Dai et al., 2019). Even if the patients survive, most of them usually live with disabilities (Pinho et al., 2019), including speech impediment, cognitive deficits, and movement disorders (Hallevi et al., 2009), in the following years. Despite ongoing research, therapies for ICH currently mainly concentrate on alleviating the primary injury and are not able to decrease the mortality (Keep et al., 2012), and effective therapies remain limited at present (Zhu et al., 2022). As a consequence, exploring therapeutic targets or novel treatments for ICH is necessary.

Artemisinin (ART) is a sesquiterpene lactone collected from *Artemisia annua* L. and serves as a widely acknowledged antimalarial drug with high levels of safety (Burkewitz et al., 2014; Tu, 2011). The fever‐reducing property of ART was first discovered by Hong Ge, a Chinese physician who was born in 283 (Efferth, 2017). Youyou Tu, the winner of the Nobel Prize in Physiology or Medicine in the year of 2015 (Chen, 2016), isolated ART (Guo, 2016; Tu, 2011) and investigated the effects in clinical trials (Tu, 2016). Alongside the well‐known effects in the treatment of malaria, ART has been demonstrated to exert multiple pharmacological effects on the central and peripheral nervous systems due to its antibacterial, antioxidant, and anti‐inflammatory properties (Zheng et al., 2016). ART is capable of protecting against free radical/oxidative stress in neurons (Gautam et al., 2009; Sarina et al., 2013). ART can decrease the death of motor neurons and the remyelination of axons to accelerate the recovery of motor function in rats after injury via reduction of oxidative stress (Chen et al., 2021). In addition, ART is able to pass through the blood–brain barrier (BBB) easily without any detectable side effects (Zuo et al., 2016), implying the pharmacological use in the treatment of neurological disorders.

Neural cell adhesion molecule L1 (L1CAM, also short for L1), a membrane glycoprotein with a molecular weight of 200—220 kDa (Rathjen & Schachner, 1984), exerts multiple essential roles in the nervous system, including roles in survival, neuritogenesis, neuronal migration, myelination, synapse formation, differentiation, axonal targeting, and synaptic plasticity (Maness & Schachner, 2007). L1 can also accelerate the regeneration in the central nervous system after lesion (Bernreuther et al., 2006; Cui et al., 2011; Roonprapunt et al., 2003; Yoo et al., 2014). L1 can affect the phosphorylation of protein kinase D1 in primary cultured mouse cerebellar granule neurons (Chen et al., 2015) and neurons in the cerebral cortex in mice with Alzheimer's disease by inhibiting oxidative stress and alleviating the neurotoxicity of oligomeric amyloid β‐protein 1–42 (Chen et al., 2020b). Berberine, a plant alkaloid with medicinal properties, enhances L1 expression in rats suffering from brachial plexus root avulsion (Chen et al., 2020a). A mimetic peptide mimicking the properties of α2,6‐sialyllactose increases L1 expression to promote neuronal survival as well as neuritogenesis (Chen et al., 2020c).

Given the protective roles of ART in the normal and diseased nervous system, the present study aimed to determine the effects of ART on ICH, focusing on its modulatory effect on L1 expression. The present study demonstrated that ART can upregulate L1 expression to reduce oxidative stress and inflammation to promote the functional recovery after ICH in mice.

## 2 MATERIALS AND METHODS

### 2.1 ICH model procedure and animal groups

C57BL/6 mice (male, weighing 25 g) were obtained from Hunan Medical Laboratory Animal Center (China). All procedures performed on mice were approved by the General Hospital of Xinjiang Military Region of the PLA Laboratory Animal Ethics Committee. Prior to generating the ICH model, all mice used in the present study were pretrained with behavioral tests for 3 d.

Mice anesthetized by intraperitoneal injection of avertin (240 mg/kg) were used to generate the collagenase‐induced ICH model as described in previous studies (Wang et al., 2020; Zhang et al., 2016) with minor modifications. Briefly, after a burr hole at the diameter of 0.15 mm was bored (along the right coronal suture at 2.0 mm lateral to the bregma), a needle with 30‐gauge (G) was inserted into the striatum at the right side (coordinates:3.75 mm underneath the dural surface, 0.26 mm anterior to the bregma, and 2 mm lateral to the midline). A total of 0.25 U collagenase IV diluted in 1 μl saline was injected into the striatum at the right side for more than 10 min, following an injection of type IV collagenase for 210 min. Aspiration was then conducted by gentle suction via a 1‐mlsyringe attached to a needle with 23‐G. This step was repeated for four times more than 15 min. The bone wax was finally applied to the seal of the burr hole, following the suture of the incision. Mice that received only needle insertion with an equal volume of normal saline served as the Sham control, without any neurological dysfunction exhibited.

To evaluate the neuroprotective role of ART in ICH, mice induced by collagenase were randomly allocated to three groups: (i) ICH, (ii) ICH + ART, (iii) ICH + ART + L1 small interfering RNA (siRNA). The groups were treated with 100 μl phosphate‐buffered saline (PBS) or ART (5 mg/kg) with or without L1 siRNA (sense: 5′‐GCA UUA GUG GCC AUC CUU UTT‐3′, antisense: 3′‐TTC GUA AUC ACC GGU AGG AAA‐5′) (Jiang et al., 2019) by intraperitoneal injection once daily.

### 2.2 Neurobehavioral function tests

#### 2.2.1 Neurologic deficit

The modified neurological severity score (mNSS) was used to assess neurologic deficits as previously described (Jiang et al., 2014) by determining exercise capability, reflexes, limb symmetry, circling behavior, balance ability, and abnormal movements, and the peak score of deficit is 18.

#### 2.2.2 Modified Garcia test

This modified test was performed on mice suffering from ICH as described. A total of seven items, including vibrissae touch, axial sensation, spontaneous activity, forelimb walking, lateral turning, climbing, and limb symmetry, were determined.

#### 2.2.3 Rotarod test

The rotarod test was used to evaluate the motor impairment in mice suffering from ICH as described previously (Zhang et al., 2016). Before and during testing, the mice placed at an increasing mode from 4 to 40 rpm within 5 min, and the time when the mouse fell off was recorded. The average value was determined based on three repeats.

#### 2.2.4 Cylinder test

The cylinder test was conducted in a transparent cylinder (10‐cm diameter and 15‐cm height). The scoring was performed as follows: percentage of the number of times of using the impaired left forelimb to contact the wall in 20 limb usages.

#### 2.2.5 Forelimb placing test

Mice with ICH were held by the trunk and located in parallel with the top of a table, allowed to brush along the surface of the table through the vibrissae on aside, and moved slowly up and down. Refractory placements for the forelimb were determined. The final score was indicated by the ratio of successful placements for the left forelimb to 10 consecutive trials ×100.

#### 2.2.6 Corner turn test

This test was carried out as previously described (Krafft et al., 2014). In the middle open side, mice with ICH were allowed to approach a 30° angle corner through 2 Plexiglas boards. The times for choosing to turn right or left were recorded. The score was indicated by the ratio of left turns to all turns ×100.

After the behavioral tests, mice were sacrificed after being anesthetized by intraperitoneal injection of avertin (240 mg/kg) to collect the tissues.

### 2.3 Neuron‐specific enolase contents

The contents present in the abdominal artery serum were determined using a commercial assay kit (H240; Nanjing Jiancheng Bioengineering Institute, Nanjing, China) according to the manufacturer's instructions.

### 2.4 Permeability of the BBB

A total of 100 μl of 4% Evans blue (Shanghai Aladdin Biochemical Technology Co., Ltd., Shanghai, China) solution diluted in saline was intraperitoneally injected. After being in circulation for 3 h, mice were anesthetized by intraperitoneal injection of avertin (240 mg/kg) and perfused transcardially. The brain was dissected and stored immediately at −80°C. After the brain at the right side was homogenized in PBS, sonicated, and centrifuged at 12,000 × *g* at 4°C for 30 min, trichloroacetic acid was added to the collected supernatant for incubation overnight at 4°C. After being centrifuged at 12,000 × *g* at 4°C for 30 min, Evans blue stain was detected at the wavelength of 610 nm using a spectrophotometer (Thermo Fisher Scientific, Inc., Waltham, MA, USA).

### 2.5 Hematoma

The collected injured brain hemisphere (except the olfactory bulb and cerebellum) was homogenized in PBS, sonicated for 60 s, centrifuged at 15000 × g for 0.5 h, and the supernatant was collected, following a 15‐min incubation with Drabkin's reagent. The absorbance was read at a wavelength of 540 nm.

### 2.6 Brain water content

The collected brain hemispheres were weighed immediately to get the wet weight. Subsequently, the brain tissues were dried for 48 h at 100°C, and then weighed again to get the dry weight. The content of brain water (%) was indicated by [(wet weight — dry weight)/(wet weight)] × 100%.

### 2.7 Tissue preparation

The collected perihematomal brain tissues were placed at 4°C overnight. Subsequently, the supernatants were harvested after centrifugation at 1000 × *g* for 15 min at 4°C. The supernatants were stored at −80°C.

### 2.8 Measurement of oxidative stress

The levels of 3‐nitrotyrosine (3‐NT), 4‐hydroxynonenal (4‐HNE), 8‐oxo‐deoxyguanosine (8‐OHDG), reactive oxygen species (ROS), glutathione (GSH), and superoxide dismutase (SOD) were determined using commercial kits (cat. nos. H394‐1, H268, H165, E004‐1‐1, A005‐1‐2, and A001‐3‐2; Nanjing Jiancheng Bioengineering Institute) according to the manufacturer's protocols.

### 2.9 Enzyme‐linked immunosorbent assay

Enzyme‐linked immunosorbent assay was performed to evaluate the tumor necrosis factor α (TNF‐α), interleukin (IL)‐1β, and IL‐6 levels as described in previous study (Wang et al., 2021) and according to the manufacturer's protocol (cat. Nos. EK0393, EK0412, and EK0526; Wuhan Boster Biological Technology, Ltd., Wuhan, China). The optical density value was determined at a wavelength of 450 nm.

### 2.10 Statistical analysis

Data are presented as the mean ± standard deviation. Statistical analyses were performed using GraphPad Prism 6 software (GraphPad Software, Inc., La Jolla, CA, USA) with two‐way or one‐way analysis of variance followed by a posthoc Bonferroni test. ^*^ *p* < .05 was considered to indicate a statistically significant difference.

## 3 RESULTS

### 3.1 ART upregulates L1 expression to improve the neurological outcome in a mouse model of ICH

To determine the influence of ART on the neurological recovery of mice following ICH, behavioral assessments, including mNSS, modified Garcia, rotarod, cylinder, forelimb placing, and corner turn tests, were performed after ICH for 24 and 72 h.

**FIGURE 1:**
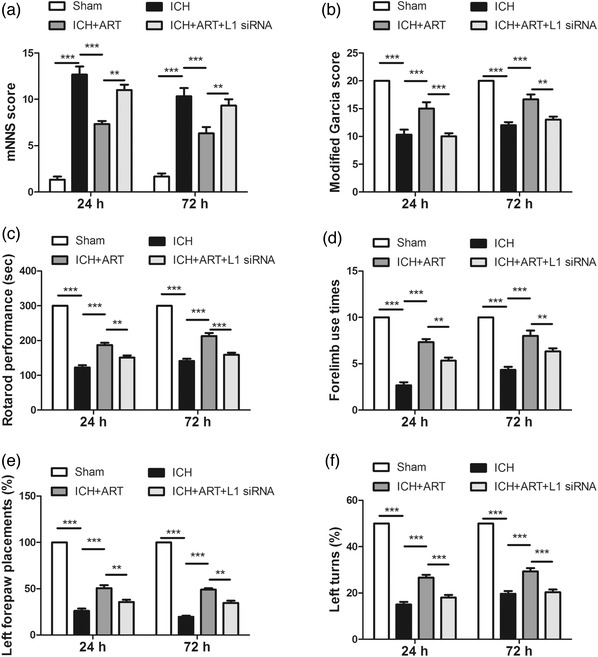
Influence of ART on the neurological outcome in mice following ICH. ART increased L1 expression to accelerate neurological functional recovery in mice induced by collagenase, as determined by (a) mNSS, (b) modified Garcia, (c) rotarod, (d) cylinder, (e) forelimb placing, and (f) corner turn tests (***p < .001, **p < .01, *p < .05, *n* = 8)

Compared with that of the sham group, the mNSS score was increased after ICH, but decreased in response to ART treatment; however, it increased when L1 siRNA was used (Figure 1(a)).

Compared with that of the sham group, the Garcia score was decreased after ICH, but increased in response to ART treatment; however, it decreased when L1 siRNA was used (Figure 1(b)).

Similar patterns to that of the Garcia score were also observed for the rotarod performance (Figure 1(c)), forelimb use times (Figure 1(d)), left forepaw placements (Figure 1(e)), and left turns (Figure 1(f)).

This suggested that ART can improve the neurological outcome in mice with ICH via upregulation of L1 expression.

### 3.2 ART upregulates L1 expression to alleviate brain edema in mice caused by ICH

To evaluate the influence of ART on ICH, the neuron‐specific enolase (NSE) content, BBB permeability, hematoma, and brain water content were determined in mice after ICH for 72 h.

**FIGURE 2:**
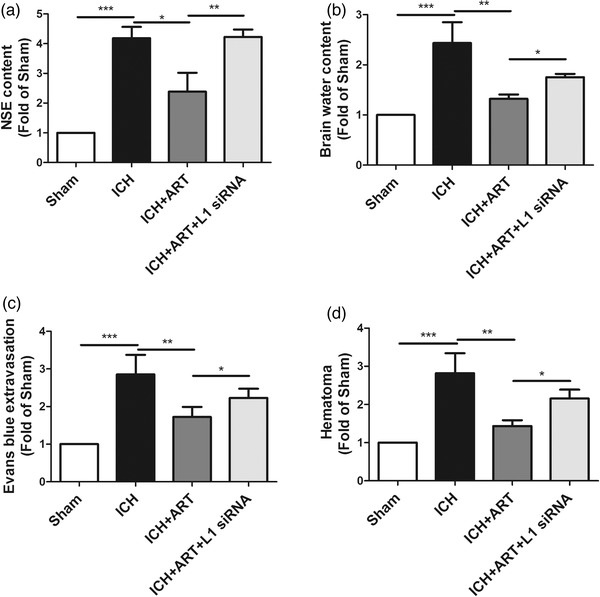
Influence of ART on brain edema in mice following ICH. ART increased L1 expression to alleviate brain edema in mice induced by collagenase, as determined by (a) NSE, (b) brain water content, (c) Evans blue extravasation, and (d) hematoma (***p < .001, **p < .01, *p < .05, *n* = 4)

Compared with that of the sham group, the NSE content was increased after ICH, but decreased in response to ART treatment; however, it increased when L1 siRNA was used (Figure 2(a)).

Opposite patterns compared with those of NSE content were observed for brain water content (Figure 2(b)), Evans blue extravasation (Figure 2(c)), and brain edema (Figure 2(d)).

This suggested that ART can reduce brain edema in mice with ICH via upregulation of L1 expression.

### 3.3 ART upregulates L1 expression to reduce the oxidative stress in perihematoma in mice caused by ICH

To detect the influence of ART on oxidative stress after ICH, oxidative stress marker levels, including 3‐NT, 4‐HNE, 8‐OHGD, ROS, GSH, and SOD levels, were determined in mice after ICH for 72 h.

**FIGURE 3:**
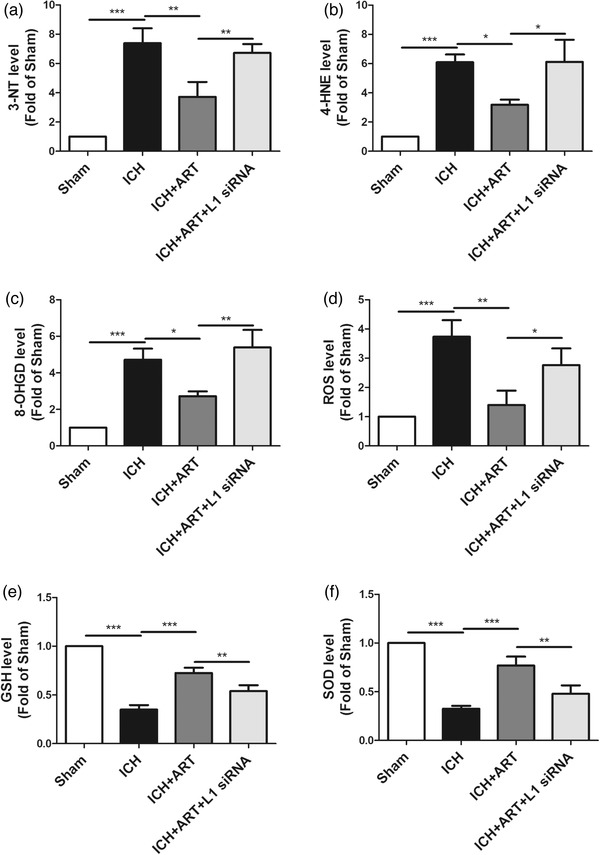
Influence of ART on oxidative stress in mice after ICH. ART increased L1 expression to inhibit oxidative stress to alleviate brain edema in mice induced by collagenase, as determined by measurements of the levels of (a) 3‐NT, (b) 4‐HNE, (c) 8‐OHGD, (d) ROS, (e) GSH, and (f) SOD (***p < .001, **p < .01, *p < .05, *n* = 4)

Compared with those of the sham group, the 3‐NT levels were increased after ICH, but decreased in response to ART treatment; however, they increased when L1 siRNA was used (Figure 3(a)).

Opposite patterns compared with those of 3‐NT levels were observed for 4‐HNE (Figure 3(b)), 8‐OHGD (Figure 3(c)), ROS (Figure 3(d)), GSH (Figure 3(e)), and SOD (Figure 3(f)) levels.

This indicated that ART could suppress oxidative stress in an ICH mouse model via upregulation of L1 expression.

### 3.4 ART upregulates L1 expression to suppress the inflammation in perihematomal in mice caused by ICH

To detect the influence of ART on inflammation following ICH, inflammation marker levels, including IL‐1β, IL‐6, and TNF‐α levels, were measured in mice following ICH for 72 h.

**FIGURE 4:**
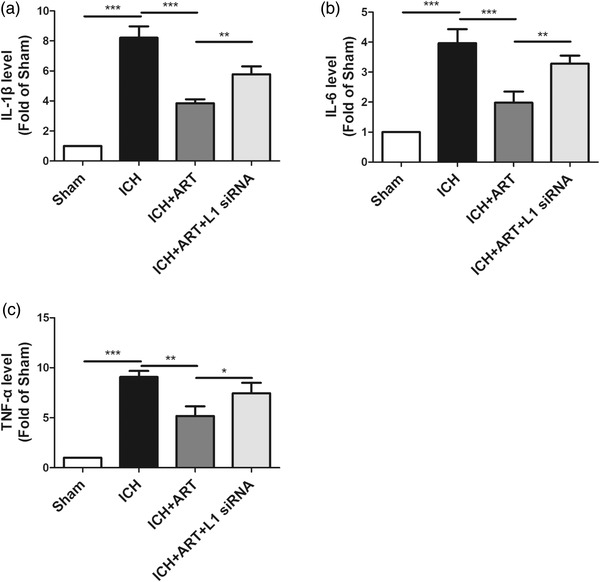
Influence of ART on inflammation in mice after ICH. ART increased L1 expression to inhibit inflammation to alleviate brain edema in mice induced by collagenase, as determined by measurements of levels of (a) IL‐1β, (b) IL‐6, and (c) TNF‐α levels (***p < .001, **p < .01, *p < .05, *n* = 4)

Compared with those of the sham group, the IL‐1βlevels were increased after ICH, but decreased after ART treatment; however, they were increased when L1 siRNA was used (Figure 4(a)).

Opposite patterns compared with those of 3‐NT levels were observed for IL‐6 (Figure 4(b)) and TNF‐α (Figure 4(c)) levels.

This indicated that ART could suppress inflammation in an ICH mouse model via upregulation of L1 expression.

## 4 DISCUSSION

The present study revealed that ART upregulated L1 expression, alleviated brain edema, reduced oxidative stress, and inhibited inflammation to alleviate ICH‐induced brain injury to improve the neurological outcome in mice after ICH. This indicated that ART may be a novel candidate treatment for ICH (Figure 4).

Collagenase was used on animals to digest blood vessel collagen and lead to bleeding into the nearest brain tissue, with pathological responses shown in human ICH (MacLellan et al., 2008). It has been widely acknowledged that neurological damage is detected at 24 h following injection of autologous blood, and the lesion is most severe at 72 h (Wang et al., 2013). Therefore, these timepoints (24 and 72 h) were selected to perform behavioral tests to determine the effects of ART on the neurological outcome of ICH in mice. The present study revealed that ART can upregulate L1 expression to improve the neurological outcome in a mouse model of ICH.

The primary and secondary brain injuries were both induced following ICH. In the initial hours of ICH onset, the primary injury is a result of the mechanical compression and destruction of perihematoma tissues by the hematoma, which causes secondary ischemia and hypoxia in adjacent tissues (Fang et al., 2019). Secondary injury is associated with brain edema, apoptosis, neuronal degeneration and necrosis, inflammatory response, and thrombin formation (Shao et al., 2019). NSE is a type of biological enzyme in the glycolysis pathway (Lamers et al., 2003; Shibayama et al., 2001) and an essential indicator of the degree of nerve injury after cerebral hemorrhage (Fizazi et al., 1998). Accordingly, hematoma volume, extent of cerebral edema, and BBB permeability can reflect the level of brain tissue damage in mice after ICH (Keep et al., 2018). The present study revealed that ART can upregulate L1 expression to alleviate brain edema in mice caused by ICH.

Oxidative stress, an important contributor for secondary brain injury after ICH due to ROS production, results in damage to proteins, lipids, and nucleic acids (Duan et al., 2016;Hu et al., 2016;Qu et al., 2016), contributing to brain edema, cell death, and further neurobehavioral deficits (Keep et al., 2012;Schlunk & Greenberg, 2015;Wang et al., 2018a). Following ICH, the oxidative products of proteins, lipids, and DNA are markedly upregulated; however, GSH peroxidase and SOD activity are downregulated (Nakamura et al., 2005;Nakamura et al., 2006).The present study revealed that ART can upregulate L1 expression to reduce oxidative stress in ICH‐induced mice.

A previous study has reported that neuroinflammation serves an essential role in the progression of brain injury caused by ICH and is characterized by an innate immune response and subsequent inflammatory cytokine production (Wang et al., 2018b). The lysis of large amounts of spilled red blood cells and inflammation activation further aggravate the BBB destruction, which promotes the formation of brain edema (Wilkinson et al., 2018). The present study revealed that ART could upregulate L1 expression to inhibit inflammation in mice caused by ICH.

## 5 CONCLUSION

The present study demonstrated that ART could accelerate the neurological functional recovery after ICH by mediating L1 expression, suggesting that ART may be developed as a novel therapeutic agent for the treatment of neurological diseases.

Although the results in this study looks promisingly, our present study is still preliminary. In this manuscript, we mainly concentrated on the effect of ART in neurological deficits in ICH mice and the L1 associated underlying mechanism. Future studies are no doubt needed to performed experiments (including TTC, IHC, etc.) to valid the effect of ART on the ICH.

### PEER REVIEW

The peer review history for this article is available at https://publons.com/publon/10.1002/brb3.2558

## AUTHOR CONTRIBUTIONS

*Conceived and designed the experiments*: J. W. *Performed the experiments and analyzed the data*: J. W., J. Y., and X. Z. *Contributed reagents/materials/analysis tools*: J. W., J. Y., and X. Z. *Wrote the paper*: J. W.

## Data Availability

The data in this study are available from the corresponding author based on the reasonable request.

## References

[brb32558-bib-0001] Bernreuther, C. , Dihné, M. , Johann, V. , Schiefer, J. , Cui, Y. , Hargus, G. , Schmid, J. S. , Xu, J. , Kosinski, C. M. , & Schachner, M. (2006). Neural cell adhesion molecule L1‐transfected embryonic stem cells promote functional recovery after excitotoxic lesion of the mouse striatum. Journal of Neuroscience, 26, 11532–11539. 17093074 10.1523/JNEUROSCI.2688-06.2006 PMC6674779

[brb32558-bib-0002] Burkewitz, K. , Zhang, Y. , & Mair, W. B. (2014). AMPK at the nexus of energetics and aging. Cell metabolism, 20, 10–25. 24726383 10.1016/j.cmet.2014.03.002 PMC4287273

[brb32558-bib-0003] Chen, S. , He, B. , Zhou, G. , Xu, Y. , Wu, L. , Xie, Y. , Li, Y. , Huang, J. , Wu, H. , & Xiao, Z. (2020a). Berberine enhances L1 expression and axonal remyelination in rats after brachial plexus root avulsion. Brain Behav, 10, e01792. 32770668 10.1002/brb3.1792 PMC7559605

[brb32558-bib-0004] Chen, S. , Jiang, Q. , Huang, P. , Hu, C. , Shen, H. , Schachner, M. , & Zhao, W. (2020b). The L1 cell adhesion molecule affects protein kinase D1 activity in the cerebral cortex in a mouse model of Alzheimer's disease. Brain Research Bulletin, 162, 141–150. 32540419 10.1016/j.brainresbull.2020.06.004

[brb32558-bib-0005] Chen, W. J. (2016). Honoring antiparasitics: The 2015 nobel prize in physiology or medicine. Biomed J, 39, 93–97. 27372164 10.1016/j.bj.2016.04.002 PMC6139675

[brb32558-bib-0006] Chen, S. X. , He, J. H. , Mi, Y. J. , Shen, H. F. , Schachner, M. , & Zhao, W. J. (2020c). A mimetic peptide of α2,6‐sialyllactose promotes neuritogenesis. Neural Regen Res, 15, 1058–1065. 31823885 10.4103/1673-5374.270313 PMC7034278

[brb32558-bib-0007] Chen, S. X. , Hu, C. L. , Liao, Y. H. , & Zhao, W. J. (2015). L1 modulates PKD1 phosphorylation in cerebellar granule neurons. Neuroscience Letters, 584, 331–336. 25445362 10.1016/j.neulet.2014.11.012

[brb32558-bib-0008] Chen, S. , Wu, L. , He, B. , Zhou, G. , Xu, Y. , Zhu, G. , Xie, J. , Yao, L. , Huang, J. , Wu, H. , & Xiao, Z. (2021). Artemisinin facilitates motor function recovery by enhancing motoneuronal survival and axonal remyelination in rats following brachial plexus root avulsion. Acs Chemical Neuroscience, 12, 3148–3156. 34465091 10.1021/acschemneuro.1c00120

[brb32558-bib-0009] Cui, Y. F. , Xu, J. C. , Hargus, G. , Jakovcevski, I. , Schachner, M. , & Bernreuther, C. (2011). Embryonic stem cell‐derived L1 overexpressing neural aggregates enhance recovery after spinal cord injury in mice. PLoS One, 6, e17126. 21445247 10.1371/journal.pone.0017126 PMC3060805

[brb32558-bib-0010] Dai, S. , Hua, Y. , Keep, R. F. , Novakovic, N. , Fei, Z. , & Xi, G. (2019). Minocycline attenuates brain injury and iron overload after intracerebral hemorrhage in aged female rats. Neurobiology of Disease, 126, 76–84. 29879529 10.1016/j.nbd.2018.06.001

[brb32558-bib-0011] Duan, X. , Wen, Z. , Shen, H. , Shen, M. , & Chen, G. (2016). Intracerebral hemorrhage, oxidative stress, and antioxidant therapy. Oxid Med Cell Longev, 2016, 1203285. 27190572 10.1155/2016/1203285 PMC4848452

[brb32558-bib-0012] Efferth, T. (2017). Cancer combination therapy of the sesquiterpenoid artesunate and the selective EGFR‐tyrosine kinase inhibitor erlotinib. Phytomedicine, 37, 58–61. 29174651 10.1016/j.phymed.2017.11.003

[brb32558-bib-0013] Fang, Y. , Tian, Y. , Huang, Q. , Wan, Y. , Xu, L. , Wang, W. , Pan, D. , Zhu, S. , & Xie, M. (2019). Deficiency of TREK‐1 potassium channel exacerbates blood‐brain barrier damage and neuroinflammation after intracerebral hemorrhage in mice. J Neuroinflammation, 16, 96. 31072336 10.1186/s12974-019-1485-5 PMC6506965

[brb32558-bib-0014] Fizazi, K. , Cojean, I. , Pignon, J. P. , Rixe, O. , Gatineau, M. , Hadef, S. , Arriagada, R. , Baldeyrou, P. , Comoy, E. , & Le Chevalier, T. (1998). Normal serum neuron specific enolase (NSE) value after the first cycle of chemotherapy: An early predictor of complete response and survival in patients with small cell lung carcinoma. Cancer, 82, 1049–1055. 9506348 10.1002/(sici)1097-0142(19980315)82:6<1049::aid-cncr6>3.0.co;2-9

[brb32558-bib-0015] Gautam, A. , Ahmed, T. , Batra, V. , & Paliwal, J. (2009). Pharmacokinetics and pharmacodynamics of endoperoxide antimalarials. Current Drug Metabolism, 10, 289–306. 19442090 10.2174/138920009787846323

[brb32558-bib-0016] Guo, Z. (2016). Artemisinin anti‐malarial drugs in China. Acta Pharm Sin B, 6, 115–124. 27006895 10.1016/j.apsb.2016.01.008 PMC4788711

[brb32558-bib-0017] Hallevi, H. , Dar, N. S. , Barreto, A. D. , Morales, M. M. , Martin‐Schild, S. , Abraham, A. T. , Walker, K. C. , Gonzales, N. R. , Illoh, K. , Grotta, J. C. , & Savitz, S. I. (2009). The IVH score: A novel tool for estimating intraventricular hemorrhage volume: Clinical and research implications. Critical Care Medicine, 37, 969–974, e961. 10.1097/CCM.0b013e318198683a PMC2692316 19237905

[brb32558-bib-0018] Hu, X. , Tao, C. , Gan, Q. , Zheng, J. , Li, H. , & You, C. (2016). Oxidative stress in intracerebral hemorrhage: sources, mechanisms, and therapeutic targets. Oxid Med Cell Longev, 2016, 3215391. 26843907 10.1155/2016/3215391 PMC4710930

[brb32558-bib-0019] Jiang, W. , Liang, G. , Li, X. , Li, Z. , Gao, X. , Feng, S. , Wang, X. , Liu, M. , & Liu, Y. (2014). Intracarotid transplantation of autologous adipose‐derived mesenchymal stem cells significantly improves neurological deficits in rats after MCAO. Journal of Materials Science. Materials in Medicine, 25, 1357–1366. 24469290 10.1007/s10856-014-5157-9

[brb32558-bib-0020] Jiang, Q. , Xie, Q. , Hu, C. , Yang, Z. , Huang, P. , Shen, H. , Schachner, M. , & Zhao, W. (2019). Glioma malignancy is linked to interdependent and inverse AMOG and L1 adhesion molecule expression. BMC cancer, 19, 911. 31510944 10.1186/s12885-019-6091-5 PMC6739972

[brb32558-bib-0021] Keep, R. F. , Andjelkovic, A. V. , Xiang, J. , Stamatovic, S. M. , Antonetti, D. A. , Hua, Y. , & Xi, G. (2018). Brain endothelial cell junctions after cerebral hemorrhage: Changes, mechanisms and therapeutic targets. Journal of Cerebral Blood Flow and Metabolism, 38, 1255–1275. 29737222 10.1177/0271678X18774666 PMC6092767

[brb32558-bib-0022] Keep, R. F. , Hua, Y. , & Xi, G. (2012). Intracerebral haemorrhage: Mechanisms of injury and therapeutic targets. Lancet Neurology, 11, 720–731. 22698888 10.1016/S1474-4422(12)70104-7 PMC3884550

[brb32558-bib-0023] Krafft, P. R. , Mcbride, D. W. , Lekic, T. , Rolland, W. B. , Mansell, C. E. , Ma, Q. , Tang, J. , & Zhang, J. H. (2014). Correlation between subacute sensorimotor deficits and brain edema in two mouse models of intracerebral hemorrhage. Behavioural Brain Research, 264, 151–160. 24518201 10.1016/j.bbr.2014.01.052 PMC3980717

[brb32558-bib-0024] Lamers, K. J. , Vos, P. , Verbeek, M. M. , Rosmalen, F. , Van Geel, W. J. , & Van Engelen, B. G. (2003). Protein S‐100B, neuron‐specific enolase (NSE), myelin basic protein (MBP) and glial fibrillary acidic protein (GFAP) in cerebrospinal fluid (CSF) and blood of neurological patients. Brain Research Bulletin, 61, 261–264. 12909296 10.1016/s0361-9230(03)00089-3

[brb32558-bib-0025] Maclellan, C. L. , Silasi, G. , Poon, C. C. , Edmundson, C. L. , Buist, R. , Peeling, J. , & Colbourne, F. (2008). Intracerebral hemorrhage models in rat: Comparing collagenase to blood infusion. Journal of Cerebral Blood Flow and Metabolism, 28, 516–525. 17726491 10.1038/sj.jcbfm.9600548

[brb32558-bib-0026] Maness, P. F. , & Schachner, M. (2007). Neural recognition molecules of the immunoglobulin superfamily: Signaling transducers of axon guidance and neuronal migration. Nature Neuroscience, 10, 19–26. 17189949 10.1038/nn1827

[brb32558-bib-0027] Nakamura, T. , Keep, R. F. , Hua, Y. , Hoff, J. T. , & Xi, G. (2005). Oxidative DNA injury after experimental intracerebral hemorrhage. Brain Research, 1039, 30–36. 15781043 10.1016/j.brainres.2005.01.036

[brb32558-bib-0028] Nakamura, T. , Keep, R. F. , Hua, Y. , Nagao, S. , Hoff, J. T. , & Xi, G. (2006). Iron‐induced oxidative brain injury after experimental intracerebral hemorrhage. Acta Neurochirurgica. Supplement, 96, 194–198. 16671453 10.1007/3-211-30714-1_42

[brb32558-bib-0029] Pinho, J. , Costa, A. S. , Araújo, J. M. , Amorim, J. M. , & Ferreira, C. (2019). Intracerebral hemorrhage outcome: A comprehensive update. Journal of the Neurological Sciences, 398, 54–66. 30682522 10.1016/j.jns.2019.01.013

[brb32558-bib-0030] Qu, J. , Chen, W. , Hu, R. , & Feng, H. (2016). The injury and therapy of reactive oxygen species in intracerebral hemorrhage looking at mitochondria. Oxid Med Cell Longev, 2016, 2592935. 27293511 10.1155/2016/2592935 PMC4880716

[brb32558-bib-0031] Rathjen, F. G. , & Schachner, M. (1984). Immunocytological and biochemical characterization of a new neuronal cell surface component (L1 antigen) which is involved in cell adhesion. Embo Journal, 3, 1–10. 6368220 10.1002/j.1460-2075.1984.tb01753.x PMC557289

[brb32558-bib-0032] Roonprapunt, C. , Huang, W. , Grill, R. , Friedlander, D. , Grumet, M. , Chen, S. , Schachner, M. , & Young, W. (2003). Soluble cell adhesion molecule L1‐Fc promotes locomotor recovery in rats after spinal cord injury. Journal of Neurotrauma, 20, 871–882. 14577865 10.1089/089771503322385809

[brb32558-bib-0033] Sarina , Yagi, Y. , Nakano, O. , Hashimoto, T. , Kimura, K. , Asakawa, Y. , Zhong, M. , Narimatsu, S. , & Gohda, E. (2013). Induction of neurite outgrowth in PC12 cells by artemisinin through activation of ERK and p38 MAPK signaling pathways. Brain Research, 1490, 61–71. 23123209 10.1016/j.brainres.2012.10.059

[brb32558-bib-0034] Schlunk, F. , & Greenberg, S. M. (2015). The pathophysiology of intracerebral hemorrhage formation and expansion. Transl Stroke Res, 6, 257–263. 26073700 10.1007/s12975-015-0410-1

[brb32558-bib-0035] Shao, Z. , Tu, S. , & Shao, A. (2019). Pathophysiological mechanisms and potential therapeutic targets in intracerebral hemorrhage. Front Pharmacol, 10, 1079. 31607923 10.3389/fphar.2019.01079 PMC6761372

[brb32558-bib-0036] Shibayama, T. , Ueoka, H. , Nishii, K. , Kiura, K. , Tabata, M. , Miyatake, K. , Kitajima, T. , & Harada, M. (2001). Complementary roles of pro‐gastrin‐releasing peptide (ProGRP) and neuron specific enolase (NSE) in diagnosis and prognosis of small‐cell lung cancer (SCLC). Lung Cancer, 32, 61–69. 11282430 10.1016/s0169-5002(00)00205-1

[brb32558-bib-0037] Tu, Y. (2011). The discovery of artemisinin (qinghaosu) and gifts from Chinese medicine. Nature Medicine, 17, 1217–1220. 10.1038/nm.2471 21989013

[brb32558-bib-0038] Tu, Y. (2016). Artemisinin—A gift from traditional chinese medicine to the world (Nobel Lecture). Angewandte Chemie (International ed in English), 55, 10210–10226. 27488942 10.1002/anie.201601967

[brb32558-bib-0039] Wang, Y. , Chen, S. , Tan, J. , Gao, Y. , Yan, H. , Liu, Y. , Yi, S. , Xiao, Z. , & Wu, H. (2021). Tryptophan in the diet ameliorates motor deficits in a rotenone‐induced rat Parkinson's disease model via activating the aromatic hydrocarbon receptor pathway. Brain Behav, 11, e2226. 34105899 10.1002/brb3.2226 PMC8413809

[brb32558-bib-0040] Wang, J. , Kuang, X. , Peng, Z. , Li, C. , Guo, C. , Fu, X. , Wu, J. , Luo, Y. , Rao, X. , Zhou, X. , Huang, B. , Tang, W. , & Tang, Y. (2020). EGCG treats ICH via up‐regulating miR‐137‐3p and inhibiting Parthanatos. Transl Neurosci, 11, 371–379. 33335777 10.1515/tnsci-2020-0143 PMC7718614

[brb32558-bib-0041] Wang, T. , Nowrangi, D. , Yu, L. , Lu, T. , Tang, J. , Han, B. , Ding, Y. , Fu, F. , & Zhang, J. H. (2018b). Activation of dopamine D1 receptor decreased NLRP3‐mediated inflammation in intracerebral hemorrhage mice. J Neuroinflammation, 15, 2. 29301581 10.1186/s12974-017-1039-7 PMC5753458

[brb32558-bib-0042] Wang, Y. C. , Wang, P. F. , Fang, H. , Chen, J. , Xiong, X. Y. , & Yang, Q. W. (2013). Toll‐like receptor 4 antagonist attenuates intracerebral hemorrhage‐induced brain injury. Stroke; A Journal of Cerebral Circulation, 44, 2545–2552. 10.1161/STROKEAHA.113.001038 23839500

[brb32558-bib-0043] Wang, G. , Wang, L. , Sun, X. G. , & Tang, J. (2018a). Haematoma scavenging in intracerebral haemorrhage: From mechanisms to the clinic. Journal of Cellular and Molecular Medicine, 22, 768–777. 29278306 10.1111/jcmm.13441 PMC5783832

[brb32558-bib-0044] Wilkinson, D. A. , Pandey, A. S. , Thompson, B. G. , Keep, R. F. , Hua, Y. , & Xi, G. (2018). Injury mechanisms in acute intracerebral hemorrhage. Neuropharmacology, 134, 240–248. 28947377 10.1016/j.neuropharm.2017.09.033 PMC6027647

[brb32558-bib-0045] Yoo, M. , Lee, G. A. , Park, C. , Cohen, R. I. , & Schachner, M. (2014). Analysis of human embryonic stem cells with regulatable expression of the cell adhesion molecule l1 in regeneration after spinal cord injury. Journal of Neurotrauma, 31, 553–564. 24125017 10.1089/neu.2013.2886 PMC3949445

[brb32558-bib-0046] Zhang, N. , Luo, Y. , He, L. , Zhou, L. , & Wu, W. (2016). A self‐assembly peptide nanofibrous scaffold reduces inflammatory response and promotes functional recovery in a mouse model of intracerebral hemorrhage. Nanomedicine, 12, 1205–1217. 26772423 10.1016/j.nano.2015.12.387

[brb32558-bib-0047] Zheng, W. , Chong, C. M. , Wang, H. , Zhou, X. , Zhang, L. , Wang, R. , Meng, Q. , Lazarovici, P. , & Fang, J. (2016). Artemisinin conferred ERK mediated neuroprotection to PC12 cells and cortical neurons exposed to sodium nitroprusside‐induced oxidative insult. Free Radic Biol Med, 97, 158–167. 27242266 10.1016/j.freeradbiomed.2016.05.023

[brb32558-bib-0048] Zhu, Y. , Huang, Y. , Yang, J. , Tu, R. , Zhang, X. , He, W. W. , Hou, C. Y. , Wang, X. M. , Yu, J. M. , & Jiang, G. H. (2022). Intranasal insulin ameliorates neurological impairment after intracerebral hemorrhage in mice. Neural Regen Res, 17, 210–216. 34100458 10.4103/1673-5374.314320 PMC8451559

[brb32558-bib-0049] Zuo, S. , Li, Q. , Liu, X. , Feng, H. , & Chen, Y. (2016). The potential therapeutic effects of artesunate on stroke and other central nervous system diseases. BioMed research international, 2016, 1489050. 28116289 10.1155/2016/1489050 PMC5223005

